# Kaposi's sarcoma of the larynx: an unusual location in an HIV-negative patient (a case report)

**DOI:** 10.11604/pamj.2020.37.206.26175

**Published:** 2020-11-02

**Authors:** Zeineb Naimi, Khalil Mahjoubi, Olfa Adouni, Rim Abidi, Maha Driss, Chiraz Nasr

**Affiliations:** 1 University of Tunis El Manar, Faculty of Medicine of Tunis, 1007, Tunis, Tunisia,; 2 Department of Radiation Oncology, Salah Azaiez Institute, 1006, Tunis, Tunisia,; 3 Department of Pathology, Salah Azaiez Institute, 1006, Tunis, Tunisia

**Keywords:** Kaposi’s sarcoma, larynx, radiotherapy

## Abstract

Head and neck involvement of Kaposi’s sarcoma is rarely encountered, especially for the Mediterranean classic subtype. Here we report a case of non-AIDS related laryngeal Kaposi’s sarcoma in a 77-year-old Tunisian man complaining of 4-month history of hoarseness and dysphagia. The patient underwent exclusive local radiotherapy with a prescription dose of 45 Gy delivered in 1.8 Gy daily fractions. He remained complaint-free for 3 months.

## Introduction

First described in 1872 as an “idiopathic pigmented cutaneous sarcoma”, Kaposi´s sarcoma (KS) is a multifocal low grade vascular neoplasm that presents most commonly in cutaneous lesions, typically located on lower extremities. Four epidemiologic-clinical subtypes of KS have been described: African-endemic, epidemic AIDS-related, classic and iatrogenic KS. Although head and neck involvement of AIDS-related KS is frequently encountered, laryngeal primary localisation is uncommon, more specifically in the classic Mediterranean form. Here we report a case of classic laryngeal KS in 77-year-old North African man treated with exclusive low dose radiotherapy.

## Patient and observation

**Figure 1:**
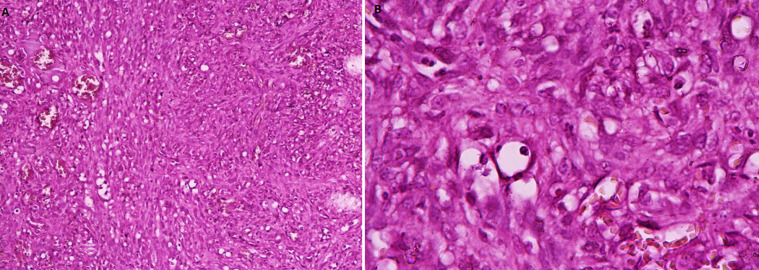
HEX Obj 20 Double mesenchymal and vascular proliferation (A) made of fascicular spindle cells in nuclei bloated with some cytonuclear atypia and dilated vessels with a thickened and hyalinized wall (B) (HEX Obj 40)

**Figure 2:**
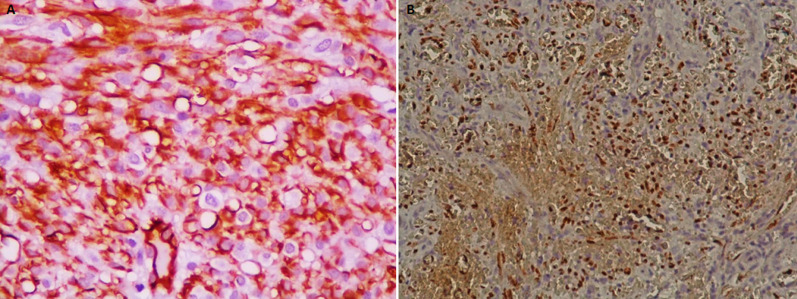
HEX Obj 40 Immunohistochemistry findings showing positive staining to CD34 (A) and HHV8 (B)

A 77-year-old Tunisian man, with past medical history of hypertension and diabetes, presented to the hospital with 4-month history of dysphagia to solids and hoarseness of voice. No dyspnoea, haemoptysis nor weight loss were reported. His history was significant for tobacco use (56 pack-year). Physical examination found multiple purple indolent macules on the left upper arm. Direct laryngoscopy revealed a massive purplish nodular lesion arising from the left pharyngo-laryngeal wall and infiltrating the left false and true vocal cords, compromising subglottis examination. Multiple biopsies were made. Due to significant oxygen desaturation, tracheostomy was performed without any complications. Histological examination of the biopsy specimen showed a double mesenchymal and vascular proliferation. The mesenchymal component was made of fascicular spindle cells in nuclei bloated with some atypical cytonuclear and few mitotic figures, while the vascular component was made of dilated vessels with a thickened and hyalinized wall (Figure 1). On immunohistochemistry examination, tumor cells were CK (-), vimentin (+), CD34 (+), CD31 (+), actine (-), desmin (-), PS100(+/-), with positive nuclear staining to Human Herpes Virus 8 (HHV8) (Figure 2). The lesion was diagnosed as a laryngeal Kaposi´s sarcoma.

**Figure 3:**
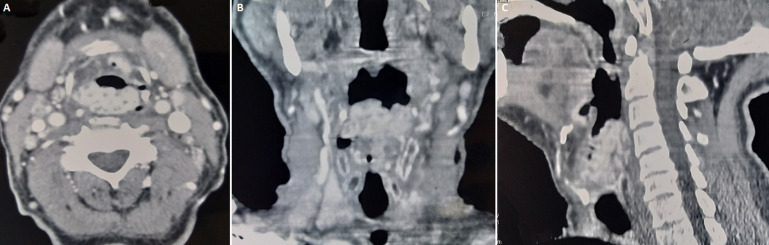
axial (A), coronal (B) and sagittal (C) CT scan images showing a large mass arising from the false vocal cord and extending to supra and subglottis

Routine blood tests showed no significant abnormalities and ELISA examination for HIV-1, HCV, and HBV were negative. Computed tomography (CT) scan revealed a large mass (4.7cmx2.3cm) arising from the left false vocal cord and extending to sub and supraglottic regions, with involvement of thyroid cartilage (Figure 3). No other visceral involvement nor lymphadenopathy were reported. Based on the head and neck multidisciplinary meeting´s decision, the patient underwent 3D conformal radiotherapy with 6 and 18 MV photon beams. The prescription dose was 45 Gy delivered in 1.8 Gy daily fractions. No prophylactic cervical lymph nodes irradiation was performed. Radiotherapy was well tolerated and the patient only experienced grade 1 acute radiation dermatitis that was well managed with topic treatment. He remained complaint-free for 3 months. The follow up was short as the patient died from pulmonary embolism 3 months after radiotherapy.

## Discussion

KS is an angioproliferative neoplasm characterized by a low-grade potential of malignancy. Although considerable progress in KS knowledge has been made, it remains unclear whether KS is a true malignant tumor or an endothelial cell reactive proliferation. HHV8 identification as the main risk factor for KS development has brought broader insight into understanding the tumor's pathogenesis. KS may manifest in four epidemiologic-clinical forms: African endemic, epidemic AIDS-related, classic and iatrogenic KS (Table 1). Histopathologically, there is no significant difference between the four subtypes. The neoplasm presents as a spindle cell proliferation with slit-like vascular spaces and inflammatory lymphocytic infiltrate. Positive nuclear staining to HHV8 is highly specific and simplifies diagnosis. As shown in our case presentation, classic KS mainly occurs in elderly patients (>70-year-old) from Mediterranean-rim countries with sex-ratio of 15:1 [1]. High incidence of classic KS has been reported in Greece, Italy North African countries and Israel. The Mediterranean KS subtype is rarely aggressive and most commonly presents in multiple cutaneous purple-reddish lesions of lower extremities associated to oedema.

**Table 1:** epidemiologic and clinical features of Kaposi’s sarcoma subtypes

	Population	Age (years)	Sex ratio	Clinical presentation
Classic	Ashkenazi Jewish and Mediterranean-rim countries	>70	15:1	Benign multifocal cutaneous purple-reddish plaques/nodules + Oedema of lower extremities
Epidemic AIDS-related	HIV-positive patients	As per primary diagnosis	100:2	Aggressive and widespread form with multifocal mucocutaneous lesions and visceral involvement
African Endemic	Central-African countries	Young adult form 25-40	17:1	Variable: benign with nodular cutaneous lesions of extremities; or florid with disseminated disease and locally aggressive lesions
		Pediatric form 2-13	3:1	Aggressive lymphadenopathic form
Iatrogenic	Immunosuppressed and organ-transplant patients	As per primary diagnosis	2.3:1	Rapidly progressive purplish plaques/nodules of extremities and oropharyngeal mucosa

Unlike the epidemic AIDS-related form, visceral involvement of classic KS is uncommon. More specifically laryngeal localisation is very rare with only few cases described in the literature [2-4]. Symptoms may include hoarseness, dysphagia, dyspnoea and cough. Cutaneous lesions are frequently associated [5]. Direct laryngoscopy with biopsies is the key examination for diagnosis. Careful attention should be paid to the risk of bleeding when proceeding to these vascular lesions´ biopsy [6]. Therapeutic options for laryngeal KS include intralesional chemotherapy, local low dose radiotherapy, laser ablation or systemic therapy for disseminated disease [7]. Several chemotherapy agents have been used such as Vincristine, Bleomycin, Vinblastine, Etoposide and Doxorubicin [8,9]. Earlier reports showed significant local regression and pain relief with low-dose local radiotherapy, with a prescription dose ranging from 20 Gy to 45 Gy, highlighting the radiosensitivity of this neoplasm [10]. Laryngeal KS may cause severe airway obstruction requiring urgent intervention. Considering the subsequent potential fatal bleeding risk, some authors recommend cricothyrotomy as an alternative approach to tracheostomy [11].

## Conclusion

Laryngeal involvement of KS is very rare, especially for the Mediterranean classic subtype. Low dose radiotherapy can offer significant symptom-relief and local regression and should be considered in the management of this localisation. Further research on targeted therapies for HHV-8 inhibition is needed, as it may provide a promising treatment for KS.
